# Down on one knee: soft tissue knee injuries across the lifespan

**DOI:** 10.1186/s13075-014-0499-8

**Published:** 2014-12-08

**Authors:** Jonas B Thorlund, Adam G Culvenor, Charles Ratzlaff

**Affiliations:** Research Unit for Musculoskeletal Function and Physiotherapy, Department of Sports Science and Clinical Biomechanics, University of Southern Denmark, Campusvej 55, 5230 Odense, Denmark; Division of Physiotherapy, School of Health and Rehabilitation Sciences, The University of Queensland, Building 84A, St Lucia, Queensland 4072 Australia; Departments of Radiology and Rheumatology, Thorne Building, Room 334D, Brigham and Women’s Hospital/Harvard Medical School, 75 Francis Street, Boston, MA 02115 USA

## Abstract

Joint injury is a potent risk factor for osteoarthritis, the most important musculoskeletal disease affecting humankind. Yet the population incidence of soft tissue knee injury is not well documented. Using health-care register data from Sweden, Peat and colleagues report that soft tissue knee injuries are common, peak in adolescence and early adulthood, have a second spike in women who are 35 to 49 years old, and continue throughout the lifespan. The study highlights the need for more knowledge on the natural history of knee injuries, their impact on knee osteoarthritis development and progression, and the potential for prevention programs to reduce the incidence of these injuries.

Joint injury is a potent risk factor for osteoarthritis (OA), the most important musculoskeletal disease affecting humankind. Although evidence is mounting that knee joint injury rates are high and increasing, it is also perhaps the lowest hanging fruit for primary OA prevention; several randomized clinical trials have shown that knee injuries can be dramatically reduced with relatively straightforward interventions. Yet outside of anterior cruciate ligament (ACL) injury and despite its potential public health impact, the population incidence of soft tissue knee injury requiring medical attention is not well documented: we have not known the extent or the nature of the problem, until now.

In a recent issue of *Arthritis Research & Therapy*, Peat and colleagues [1] provided population-wide estimates of clinically diagnosed soft tissue knee injuries across all ages on the basis of an entire region of Sweden (approximately 1.3 million people). The opportunity to report and classify all clinically diagnosed knee injuries across the lifespan arises from unique and detailed health-care registries typical to Scandinavian countries. This overcomes weaknesses of previous epidemiological evaluations of knee injuries, which are limited to specific health-care settings, subgroups of people, and specific injury types. Of note, the findings of Peat and colleagues [1] have convergent validity - largely agreeing with previous reports of incidence for specific injury types and subgroups where data overlap.

What emerges is that population exposure to soft tissue knee injury is a common problem; the annual incidences for males and females are 766 and 676 per 100,000 persons per year, respectively. This is approximately 10 times higher than ACL injuries alone. If these ‘less catastrophic’ but more common injuries are a risk for OA development (as risk factor studies measuring self-reported injury suggest [2]), then this study may be uncovering and detailing critical new exposure data. They are clearly more numerous though more difficult to accurately diagnose. This study begins to shed light on this challenge.

Also revealed is new information on age and gender differences. The incidence of soft tissue knee injuries peaks in adolescence and early adulthood and is likely sports-related, matching seasonal fluctuations in popular sports in Sweden. The rates after this period decline over the lifespan with a notable exception: females from 35 to 49 experience a second peak. This is intriguing and the reasons are not clear, although the authors propose that the previously reported link between parity/child-bearing and knee OA may be mediated by injury. Although the reasons remain obscure, the finding is compelling and may help elucidate the consistently reported, but unexplained, higher prevalence of knee OA in females.

Peat and colleagues [1] show that, although incidence rates are highest in the second and third decades of life, considerable rates of contusion, collateral ligament sprain, and other soft tissue strains continue into middle and old age. These injuries coincide with the age of onset of knee OA symptoms and illustrate the challenge of differentiating what is truly an injury from what is part of a previously latent or degenerative process or both. This also applies to meniscal injuries. Surgeries for meniscal tears peak in the mid to late 40s [3–5]. In contrast, Peat and colleagues [1] report a high incidence of meniscal tears in adolescents and young adults. As acknowledged by the authors, less severe injuries such as meniscal tears likely suffer from some misclassification. However, the relationship between diagnosis and surgery for meniscal tears requires further investigation.

The high injury incidence among adolescents and young adults, together with the known risk of OA incidence from ACL and meniscal injuries, provides further impetus for implementing knee injury prevention programs, for which there is a strong body of level 1 evidence [6–11]. Efficacy has been demonstrated primarily in the sports team setting, implemented as novel 10- to 15-minute team warm-ups consisting of neuromuscular exercises to train athletes to land, decelerate, and push off with better lower limb alignment and improved trunk control, balance, and proprioception. The reported risk reductions range from 41% to 88% [7,8,11]. Given the age and frequency at which these injuries most often occur and their potential sequelae, perhaps targeting injury prevention programs to physical education classes in public schools could address a growing public health problem.

The study by Peat and colleagues highlights several areas for further study. Knowledge is needed on the natural history of knee injuries in the development of knee OA as well as the potential for prevention programs to reduce the incidence. The spike of injuries in females between 35 and 49 requires confirmation and further investigation as to its causes, prevention, and potential role in OA development or progression. The same is true for injuries that occur in middle and older age, often coinciding with a time when knee OA has been diagnosed. Further clarity is needed around meniscal injury: what is traumatic injury and what is degenerative knee disease? There is still much to discover about the different knee injury types throughout the lifespan and the initiation and progression of knee OA. The study by Peat and colleagues [1] provides a good platform for this to be pursued.

## Abbreviations

ACL: Anterior cruciate ligament
OA: Osteoarthritis
